# The Arabic Nurse Professional Competence-Short Version Scale (NPC-SV-A): Transcultural Translation and Adaptation with a Cohort of Saudi Nursing Students

**DOI:** 10.3390/healthcare11050691

**Published:** 2023-02-26

**Authors:** Mohammad Hamdi Abuadas

**Affiliations:** Nursing Faculty-Khamis Mushait, King Khalid University, Abha 61421, Saudi Arabia; mabuadas@kku.edu.sa

**Keywords:** nursing students, clinical competence, psychometric properties, cultural adaptation

## Abstract

Background: There is broad consensus that assessing and improving the competence of nurses is a crucial element of nursing education and practice. Numerous national and international nursing research studies have used the 35-item Nurse Professional Competence Scale (NPC-SV) to measure the self-reported competence of nursing students and registered nurses. To increase its usage in Arabic-speaking nations, however, a culturally adapted Arabic version of the scale with the same level of quality was necessary. Objectives: The study aimed to develop a culturally adapted Arabic version of the NPC-SV and evaluate its reliability and validity (construct, convergent, and discriminant types). Methods: Methodological descriptive cross-sectional design was utilized. A convenience sampling technique was employed to recruit 518 undergraduate nursing students from three Saudi Arabian institutions. The translated items were appraised by a panel of experts, who considered the content validity indexes. The structure of the translated scale was examined using exploratory and confirmatory factor analysis, structural equation modelling, and the Analysis of Moment Structure method. Results: When utilized with nursing students in Saudi Arabia, the Arabic short version of the Nurse Professional Competence Scale (NPC-SV-A) was shown to be reliable and valid in terms of its content, construct, convergent, and discriminant validity. Cronbach’s alpha for the entire NPC-SV-A scale was 0.89 and varied from 0.83 to 0.89 for each of the six subscales. Exploratory factor analysis (EFA) revealed six significant factors with 33 items that accounted for 67.52 percent of the variance. The scale was congruent with the suggested six-dimensional model, as confirmed by confirmatory factor analysis (CFA). Conclusion: The Arabic version of the NPC-SV, which was reduced to 33 items, showed good psychometric properties, with a six-factor structure accounting for 67.52% of the total variance. When used alone, this 33-item scale can allow for more in-depth evaluations of self-reported competence among nursing students and licensed nurses.

## 1. Introduction

Nursing competency extends beyond skills and knowledge. It is also an aspect of being able to perform complex tasks through the deployment and utilization of psychological resources, which include abilities, beliefs, and practices, in a specific context [1]. It is vital to assess clinical competency since patient safety and quality of care are interdependent on the nurse’s clinical competence [2]. Professional certification and a Bachelor of Science degree are awarded to nursing students in Saudi Arabia once they have completed a four-year program and a year of internship. Rather than having doctors and other medical experts handle much of the teaching, nursing schools are adopting a competency-based curriculum. The Education and Training Evaluation Commission (ETEC) oversees the national accrediting system, while the Saudi Commission for Health Specialties (SCFHS) monitors nursing programs. Clinical rotations are conducted at teaching hospitals under the guidance of university professors or ward nurses, just as they are in other countries [3]. Efforts should be made to replace passive, teacher-centered learning with interactive, student-centered learning, which has been demonstrated to improve students’ comprehension of the nursing profession and their ability to acquire necessary skills [4]. Since nursing students are at the epicenter of these shifts, it is imperative to evaluate their self-reported clinical competence to ascertain if they are adequately prepared to assume their future professional roles. This underscores the necessity for a reliable and valid tool for evaluating nursing students’ professional competence based on their core skills. The use of a valid and reliable research tool is greatly valued [5]. The presumption that it permits results comparisons across many studies from both international and domestic research is one justification for this [5,6]. Another presumption is that using validated tools raises the confidence that the tools correctly represent what they are meant to measure [5,6]. A previously validated instrument does not, however, always imply that it is valid in a different period, culture, or situation [5,6,7]. When an instrument is utilized in a new language, place, or time, the cross-cultural adaption process is crucial to lowering the risk of research bias [6]. Additionally, perceptions cannot be directly quantified [7]. As a result, perceptions are inferred from a collection of survey items and measured indirectly [7]. It may be difficult to compare results across cultures and groups in research when a phenomenon is monitored indirectly via surveys. If the adaption process was incorrect, comparison will be challenging. It is crucial that each item be carefully adjusted.

## 2. Background

There is widespread agreement that evaluating and enhancing nurses’ competence is a crucial part of nursing education and practice. Many people all around the world have tried to figure out how to measure a nursing student’s progress through school, and many different tools have been created to do so [4]. In the international literature, various valid and reliable tools are available to evaluate nurses’ competencies. Most of the emerging instruments are grounded in either a literature study [8] or a conceptual analysis [9]. The only scale that is consistent with the six core competencies identified by the Institute of Medicine is the Nurse Professional Competence (NPC) scale, which was developed in 2014 and revised in 2018 by Swedish experts [10]. This scale was based on formal competence requirements from the Swedish Society of Nursing (2017) and the World Health Organization (2001) [11,12]. Several studies found the NPC scale, which had 88 items organized into eight categories, to be psychometrically sound [10,13,14,15]. In addition to Austria, Germany, Norway, Portugal, Switzerland, and Australia [16], this English translation of the NPC scale [17] has also been used to evaluate samples of clinical nurses [18] and nursing students who are about to graduate [15]. The original NPC scale was time-consuming for responders due to the 88-items long scale. To increase the response rate, the NPC scale’s short version (NPC-SV), which has 35 items, was designed [10]. The NPC-SV examined six competency domains: delivered nursing care; value-cantered nursing care; techno-medical care; pedagogy in nursing care; nursing care documentation & administration; and nursing care innovation, administration, & management. Cronbach’s alpha values for the NPC-SV ranged from 0.71 to 0.86, indicating its acceptable reliability. Confirmatory factor analysis using principal components analysis, and the scale’s prior studies’ known-group validity all supported the scale’s construct validity [10,18]. Challenges and concerns are raised for the standards of nursing practice and their delivery while dealing with an Arab patient due to the significant differences in social and religious characteristics of the Arab patient. Problems that go beyond linguistic barriers and might involve divergent views on topics like illness, treatment, and even death [19]. Nurses and medical professionals need to have a well-rounded knowledge of the many cultures that their patients may have. Providing a holistic level of care for patients necessitates taking into account their individual preferences and needs, as well as the norms and values of their respective social cultures [20]. One’s ability to counsel patients of Arab descent on appropriate medical treatment and establish a rapport of trust depends on one’s familiarity with the cultural norms and values of their community [21].

It is not uncommon for cultural barriers to arise between Western care providers and care providers of Arab heritage and the patients they are dealing with [19]. Cultural differences, differences in communication styles, and the language barrier all contribute to tense interactions [19]. Arabs may seem different on the outside, yet they act and think similarly. Things like having a strong sense of family unity and having a positive perspective on illness and health can play a role here. Health care providers face a wide range of challenges, from gathering sufficient information to conveying the importance of the recommended behavior change to patients. There should be translations into languages and cultures where the target audience resides in order to fully comprehend the unique experiences of Arab and Middle Eastern patients [19]. The inclusion of cultural sensitivity training in both fundamental nursing studies and professional nursing practice is becoming more expected [19]. Individual nurses and the nursing community as a whole are coming to see the need for transcultural nursing training and certification in order to safeguard against future ethical and legal issues and better serve patients from a wide range of cultural backgrounds [19]. The academic and clinical instructors at schools of nursing are the primary influencers on students’ attitudes and behaviors as professionals, hence, an increasing number of nursing academics are emphasizing cultural competency of research techniques as a necessity for nursing research excellence [19,21]. Over the past three decades, the importance of nursing schools preparing staff and educating students to perform culturally competent research that contributes to better care for diverse populations has been increasingly apparent [19]. Key to determining the true state of the phenomenon being studied is the availability of transculturally adapted instruments that consider the cultural differences of the target group [9,21]. There is a dearth of research that examines nurses’ perceptions of their clinical competence or the factors that contribute to nurses’ overall clinical competence in Saudi Arabia. Use of culturally appropriate Arabic scale that is reliable and valid is essential for such research. Therefore, the aims of this research study were to develop a culturally adapted Arabic version of the NPC-SV and to evaluate its psychometric properties.

## 3. Methodology

### 3.1. Design

In the current study, the NPC-SV scale was translated, and its Arabic version was validated among Saudi nursing students as part of a descriptive cross-sectional methodological research [22].

### 3.2. Sample and Setting

All nursing students enrolled in a bachelor’s degree program at a Saudi Arabian university constituted the study population. In this study, a sufficient sample size was achieved using convenience sampling. Five hundred eighteen nursing students from three government institutions in the northern, southern, and central regions of Saudi Arabia were recruited. The sample size was calculated based on the guidelines provided by Boateng et al. [5], who suggested a subject-to-item ratio of at least 10:1, or 200 subjects. Thus, a minimum sample size of 350 subjects was determined to be required based on the 35-item scale. The inclusion criteria were being enrolled in the bachelor’s degree in nursing at Saudi Arabian University; being in the third, fourth, or internship year of study; and agreeing to participate in the study. Students in their first or second year of studies were not permitted to participate. Data collectors contacted potential participants and evaluated their eligibility. Requests were made to data collectors to deliver questionnaires to people who met the inclusion criteria.

### 3.3. Data Collection

The data collection instruments were given to the subjects (nursing students in their 3rd, 4th, and internship year), via the designated data collectors, after acquiring the appropriate approvals to conduct the research. Prior to collecting data, permission from colleges was acquired. Data was gathered from the months of October 2021 through March of 2022. Those who consented were given the questionnaire in their classrooms or clinical settings. Once that was completed, the study’s goals were communicated to the participants. As participants were given adequate time to fill out the questionnaire, the data was gathered at the same location. It took roughly 15 min to complete the questionnaire.

### 3.4. Ethical Considerations

Prior to data collection, institutional review board Approval (IRB) was acquired by the Ethical Committee of Scientific Research (Approval No. (HAPO-06-B-001) (ECM#2019-80-2). Each questionnaire came with a cover letter and consent form that explained everything about the research in depth. Individuals who agreed to participate in the study did so voluntarily and were assured complete confidentiality. Each participant provided their agreement after being fully informed of the risks involved.

### 3.5. Scale Translation and Cultural Adaptation

Firstly, translations of the NPC-SV scale were made into Arabic and validated against the English originals using Brislin’s model [6,7]. Two bilingual specialists independently translated the scale into Arabic and came to comparable results. The two bilingual specialists then gathered, went through the translations as a group, and decided on the initial version of the translated scale. Thereafter, another two bilingual experts independently back translated the synthesized Arabic scale version into English [6,7]. The original meaning of the English version was captured by the two back translations, which were nearly identical. Furthermore, the back-translation did not lead to any suggested phrase changes.

Secondly, the cross-cultural compatibility and translation were evaluated by a panel of four Saudi multilingual health care experts. The panel evaluated the link between the scale’s underlying domains in both the original and target contexts [21]. In addition, the panel evaluated whether the instrument’s modified items are as relevant and acceptable in the target population as they were in the original population [21]. The panel suggested a few wording modifications. The translated scale was revised in line with the suggested adjustments, and the qualified experts decided that the Arabic version of the revised scale, as shown in Appendix A, was culturally appropriate. As the panel replaced the concept of relatives with that of the family, it’s apparent that they gave the concept of family more weight than that of relatives. The family, not the individual, is the center of Arab society, and it is inside the home that children are first introduced to the culture’s norms and customs. The importance of family, respect for elders, devotion to one’s kin, and fulfilling one’s duties are among the most important principles for an Arab culture.

### 3.6. Content Validity

Two phases of reviews were conducted by mailing the Arabic culturally adapted version of NPC-SV to a panel of six nursing education experts to evaluate the content validity index for total scale (S-CVI) and for scale items (I-CVI) [22]. In the first phase, experts were provided with a list of scale items as well as the theoretical and operational meanings of scale constructs so that they could offer suggestions on how to best phrase each item. The Arabic version of NPC-SV was updated and improved based on the feedback and recommendations provided. In the second phase, the relevance of the updated Arabic version of NPC-SV was evaluated by specialists using a 4-point ordinal scale: 4 = extremely relevant, 3 = fairly relevant, 2 = slightly relevant, and 1 = not relevant. Using Lynn’s (1986) technique, an item’s content validity is considered satisfactory if its item-level content validity index (I-CVI) is greater than 0.78, and its scale-level content validity index (S-CVI) is greater than 0.80 [23]. On the basis of these findings, we may conclude that all items on the CHBM-PCS and the entire scale have good content validity. Both the S-CVI (0.90) and I-CVI (0.81–0.99) exceeded the cut-off value of 0.78, while the S-CVI (0.90) was also over the cut-off value of 0.80. These percentages point to the fact that the items on the scale are indeed distinguishable with regards to the constructs under study.

### 3.7. Data Analysis

The IBM Statistical Package for Social Sciences (SPSS) software (version 21.0) and Analysis of Moment Structure (AMOS) (version 21.0) tools were used for all data analyses. The data collected was summarized using descriptive statistics. Calculating the mean (M), standard deviation (SD), and adjusted item-total correlation were part of the item analysis. Using the internal consistency procedure, the Arabic version of the NPC-SV scale’s reliability was assessed. Both exploratory and confirmatory factor analysis were used to evaluate the construct, convergent, and discriminant validity. We confirmed normality, independence, and homoscedasticity before model testing. The missing data that emerged at random was replaced using the case-mean imputation technique. Using the cut-offs recommended by Morin et al. [24], the model fit was assessed. The following criteria must be met: (1) a critical ratio of factor loadings more than 1.96, (2) relative chi-square less than 5, (3) the normed fit index and the comparative fit index more than 0.85, (4) adjusted goodness of fit index and the goodness of fit index more than 0.85, (5) the standardized root mean square residual and root mean square error of approximation less than 0.08 [24].

## 4. Results

### 4.1. Socio-Demographic Characteristics

A total of 462 nursing students participated in the survey, yielding an 82% response rate. The mean age was 21.96 years (SD 0.98), and it ranged from 21 to 24 years. Among the students included in the sample, females made up over 60% (61.96%), while males made up less than 40% (38.03%). With respect to year of study, 36.48% were interns, 33.39% were in their fourth year, and 30.11% were in their third year. Clinical training was completed by students on medical floors (35.91%), surgical floors (32.23%), and specialty wards (31.85%). With respect to the length of clinical training, 24.13% undergo clinical training for less than 4 weeks, 38.22% for a period of 4–7 weeks, and 37.64% for more than 7 weeks.

### 4.2. Perceptions of Professional Clinical Competence

In general, students gave an average of 80.4 on the NPCS-SV scale (SD = 8), indicating a high level of satisfaction with their own professional competence. Total mean scores (M = 82.8, SD = 8) were higher for male students than female students (M = 77.9, SD = 9). Students in their internship year had the highest mean total score (M = 85.6, SD = 9), followed by those in their third and fourth years of nursing school (M = 76.9, SD = 8 and M = 78.6, SD = 8, respectively). Overall, students who received their training on the medical or surgical floors gave the clinical environment comparable ratings (M = 77.9, SD = 9 and M = 78.8, SD = 8, respectively), whereas those who received their training in the specialized units provided a higher rating (M = 84.3, SD SD = 8). (See Table 1)

### 4.3. Construct Validity

The 35 items from the Arabic version of the scale (NPC-SV-A) were aggregated, and EFA was utilized to determine their factor structure. The factors were identified using a principal component analysis technique. Using the Varimax method, the resulting factors were rotated such that they were orthogonal to the original set. Kaiser–Meyer–Olkin and Barlett tests were used to determine whether the sample size was large enough before the EFA data were analyzed. Before evaluating the EFA findings, the Kaiser–Meyer–Olkin and Barlett tests were used to check the appropriateness of the sampling. According to the results, two items were eliminated: item no. 3 and item no. 19. The following causes led to the deletion of these items: (i) factor-item loadings were less than 0.40, and (ii) factor-item loading was not clean; factor-item loading is deemed clean if the absolute difference between loadings is less than 0.20 [5]. Then, only those items were maintained that best mirrored the theoretical dimensions (constructs) outlined previously. The 33-item NPC-SV-A scale was therefore re-evaluated by EFA utilizing the same analytical approach and rotation process. The Kaiser–Meyer–Olkin value was 0.96, and the findings of the Barlett test were highly significant (χ^2^ = 19,783.37; df = 1398; *p* < 0.001). All factors with eigenvalues less than one were retained [5]. The EFA results supported the number of anticipated factors (six significant factors). All items on each factor belonged to the same construct, and the total variance explained by all factors was 67.52 percent.

The eight items comprising the Nursing care administration and documentation subscale loaded as Factor 1 and explained 15.91% of the variance. Factor 2 accounted for approximately 14.02 percent of variation and comprised all six items of the subscale for Techno-medical care. Factor 3 comprised all six items of the Nursing care innovation, administration, and management subscale, accounting for 12.65% of the variance. Factor 4 accounted for about 10.77% of the variation and reflected each of the subscale’s five items. Factor 5 produced all four pedagogies in nursing care subscale items and explained approximately 8.35% of the variation. Factor 6 accounted for about 5.82% of the variation and comprised all four subscale items of delivered nursing care. In the rotated pattern matrix, loadings varied from 0.638 to 0.871. (See Table 2)

**Figure 1:**
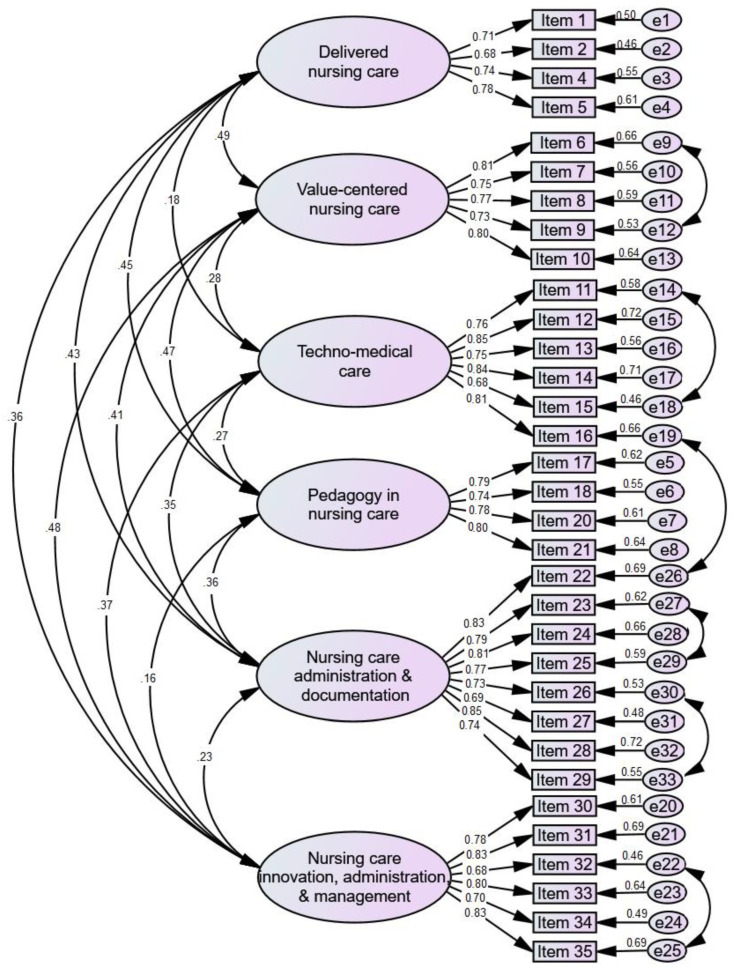
Schematic diagram showing the CFA of standardized regression for NPC-SV-A items (*n* = 518).

Confirmatory factor analysis (CFA) was used to examine the underlying dimensions of the NPC-SV-A scale. The findings indicate that the model reflecting six correlated factors was the most acceptable for the 33-item NPC-SV-A scale. The schematic diagram of the CFA of standard regression for 33 items of the NPC-SV-A scale is depicted in Figure 1. The construct validity of the six-factor model retrieved from EFA was evaluated using CFA and the maximum likelihood estimation technique. The criteria for evaluating the model’s fit were based on the cut-offs for acceptable fit given by Byrne [25]: (i) the index of relative chi-square (χ^2^/df) should be less than 4, (ii) a critical ratio (CR) more than 1.96, (iii) the goodness of fit index (GFI) and adjusted goodness of fit index (AGFI) should be more than 0.85, (iv) the standardized root mean square residual (RMR) and root mean square error of approximation (RMSEA) should be less than 0.08 (v) the comparative fit index (CFI) and the normed fit index (NFI) should be more than 0.85 [25]. All factor loadings ranged from 0.68–0.87 in this investigation. The CR for all factor loadings was >1.96. GFI = 0.87, χ^2^ = 2731.04, df = 1240, χ^2^/df ratio = 2.20, *p* < 0.001, CFI = 0.93, AGFI = 0.85, and RMSEA = 0.05 were the fit indices for the 33-item NPC-SV-A scale. The outcomes confirmed that the model fits the data adequately.

### 4.4. Convergent and Discriminant Validity

When completing a CFA, it is essential to demonstrate convergent and discriminant validity [5]. Convergent validity was determined by comparing each subscale’s average variance extracted (AVE) to its correlations with the other subscales; when AVE was greater than 0.50 and greater than the subscale’s correlations with the other subscales, convergent validity was established [5]. Maximum shared variance (MSV) and average shared variance (ASV) should be less than AVE for all subscales to establish discriminant validity [5]. Subscales intercorrelations and measures of convergent and discriminant validity are shown in Table 3. All AVE values for the NPC-SV-A subscales were more than 0.50, demonstrating that the NPC-SV-A scale has convergent validity. Evaluation of MSV and ASV, both of which were shown to be lower than the AVE for all subscales, allowed for the establishment of the discriminant validity of the NPC-SV-A scale. Convergent and discriminant validity analysis was conducted using the Master Validity Tool plug-in developed by Gaskin and Lim [26].

### 4.5. Reliability

Internal consistency tests were used to establish the reliability. The reliability of the NPC-SV-A was calculated using Cronbach’s alpha values for both the whole scale and each of its individual subscales. Scales with a Cronbach’s alpha of 0.70 or above are generally accepted as reliable. Scales with an alpha of 0.80 or higher show very good internal consistency [5]. Furthermore, the eligibility guidelines were used to identify dysfunctional items: (1) a correlation of <0.30 between an item and the subscale score, or (2) an increase of >0.10 in the overall scale reliability when the item was eliminated [5]. All six subscales had good Cronbach’s values, which were independently determined to be between 0.83 and 0.89. All item-total correlations after correction were found to be more than.30, falling within the range of 0.60 to 0.78. As the results of the item analysis did not indicate that the reliability of the scale would improve noticeably without any particular item, no items were removed. The overall Cronbach’s alpha value for the NPC-SV-A scale was 0.89.

## 5. Discussion

The primary goal of this research was to evaluate the cultural appropriateness and reliability of a shortened version of the Nurse Professional Competence Scale that had been translated and modified for use in the target population. Based on the results of this research, it can be concluded that the NPC-SV-A is a valid and reliable instrument for assessing professional competence in Saudi nursing students. It was determined that there was internal consistency and independence between each of the NPC-SV-A subscales. All subscales had high levels of internal consistency, as shown by Cronbach’s alpha values between 0.83 and 0.89, and all subscale items had satisfactory adjusted item correlations of >0.30; they ranged from 0.60 to 0.78. This study produced greater reliability coefficients than Nilsson, Engström, Florin, Gardulf, and Carlsson [10], whose alpha coefficients were lower, yet acceptable. In addition, the reliability coefficients found in this investigation were highly comparable to those reported in a prior Chinese study [27]. Future research should involve more testing of these scales. These findings were lower than those reported in the English version of the NPC-SV, which was assessed among registered nurses in Saudi Arabia, with Cronbach’s alpha values for the variables of 0.86–0.93 [18]. It appears that both the item-level and overall content validity of the NPC-SV-A are adequately high. The overall S-CVI of 0.92 was within acceptable range, whereas the I-CVIs of the individual items varied from 0.81 to 0.99. These scores demonstrated that the items on the scale are distinctively relevant to the underlying constructs. The content validity indices have been reported in previous studies. Both exploratory and confirmatory factor analysis were used to examine the construct validity since they reflect distinct conceptual processes for determining validity. The six measures were all found to be unidimensional using both exploratory and confirmatory factor analysis. EFA results showed that the item factor loading was acceptable, with values of 0.64–0.87. Item 3 (Satisfying the patient’s confidential physical needs) is an exception, with a loading to factor 3 (delivered nursing care) of 0.31, which does not meet the item retention criteria. The factor loading values for item 3 may have been poor due to redundancy or similarity of meaning with item 2 (Satisfying the patient’s physical needs). In addition, item 19 (Inform and educate patient groups and families) was deleted due to poor item loading. This could be attributed to the fact that item 18 (Inform and educate patients and families) has quit similar linguistic meaning and construction. In sum, the 33 items of the NPC-SV-A strongly suited the data and supported the original scale structure, according to the CFA findings, which were also obtained. Both exploratory and confirmatory factor analyses revealed that each of these items satisfied the loading threshold and loaded strongly on each factor, providing compelling statistical support for the construct validity of the six subscales. Xu, Nilsson, Zhang, and Engström [27] showed similar results, in which the majority of selected items loaded significantly on their respective subscales in both exploratory and confirmatory factor analysis. However, the Arabic version has a reduced set of items. Student perceptions of clinical competence varied by gender, academic year, and training ward, according to the study’s interesting findings. The demographics of the students in this study were comparable to those of students assessed in previous studies [2,8] on their views of their own clinical competence as a student. This study, one of the few that has evaluated nursing students’ clinical competency in Saudi Arabia, found a wide variety of obstacles to students’ capacity to transfer their skills into real-world settings. There was also no statistically significant variation in how students at multiple universities judged their own levels of competence, according to the research. Perhaps this is because Saudi Arabia has put in such great effort to provide access to undergraduate education that attendance at the country’s public institutions is nearly universal. Students in Saudi universities had optimistic views of their learning competence [15,16,18], echoing findings from a previous study using the NPC-SV. Higher-than-anticipated student ratings were found. The exceptional quality of clinical education given by Saudi colleges, which benefits from simulation labs and a variety of teaching resources, may help explain this pattern (such as library services and facilities and nursing simulation laboratory equipment). Moreover, Saudi Arabia has created training hospitals and a state-of-the-art healthcare system. Students may have adapted their settings to be more productive in this setting, and the findings may also suggest that students find satisfaction in education no matter where it takes place. This study demonstrated that students’ perceptions of the clinical training improved as they advanced in their clinical training. Similar outcomes have been obtained by another research using the NPC-SV [15,17]. One explanation for these findings is that Saudi students generally judge clinical competence less positively in their first years of study, when the experiences are novel, and that their judgments change as they become more accustomed to and confident in the clinical environment, as well as they become more accustomed to and confident in themselves. Additionally, as students become more used to and confident in the academic environment, their judgements change.

The perception of clinical competence was greater among males and those educated in specialized units. With the limited number of studies performed under similar conditions, Ramsbotham et al. [28] found similar findings. Furthermore, student nurses’ academic experiences have been found to have a significant impact on their development as clinical professionals [18]. These results indicate that the NPC-SF-A is a valuable tool for distinguishing between the professional competences of nursing students with varying levels of education. Further testing and refinement of the Arabic NPC-SV is recommended, especially with regard to the subscales measuring nursing care delivery and nursing pedagogy. Results might be strengthened by repeating the study with the same or a comparable demographic, or even with people from different backgrounds. Prospective studies can add to validation of the Scale.

This study has a number of limitations. First, the stability of the scale over time was not determined since repeated surveys were not undertaken. Cross-validation studies are necessary to verify the reliability and validity of the NPC-SV-A scale in various populations and to confirm the component structure of the NPC-SV-A scale. Thirdly, the sample may not have been representative due to the participants’ self-selection, which may have included individuals with a stronger interest in the issue. In addition, further study is necessary to explore variations in organizational culture and the clinical competency scale between nations. Lastly, information on the interpretation of scale scores should be accessible; along with an indication of what score change constitutes a minimum significant change.

## 6. Conclusions

When utilized with Saudi nursing students, the Arabic version of the NPC-SV was shown to be reliable and to have satisfactory content, construct, convergent, and discriminant validity. It may be used by student nurses and professional nurses to evaluate the clinical competency of Saudi nursing students. Such an evaluation is required to determine the level of clinical competence and to design training programs that are especially matched to the clinical environment of the students. The scale may also be used to evaluate the efficacy of intervention initiatives. In addition, the scale may be useful for many Arabic-speaking communities. Due to the diversity of languages and cultures within the Arab globe, the word implications of the scale items should be evaluated prior to their usage in other Arab nations.

## Figures and Tables

**Table 1:** Reliability Coefficients for Nurse Professional Competence Scale-Short Version (NPC-SV) Subscales and students’ overall mean score by sex, academic year, and training unit type (*n* = 518).

Factors	Number of Items	Overall Mean (SD) (*n* = 518)	Cronbach’s Alpha	Range of Corrected Item-Total Correlations	Sex		Academic Year			Training Unit		
					Female (*n* = 321)	Male (*n* = 197)	Interns (*n* = 189)	Academic Year 4 (*n* = 173)	Academic Year 3 (*n* = 156)	Medical Units (*n* = 186)	Surgical Units (*n* = 167)	Specialized Units (*n* = 165)
Value-centered nursing care	5	12.3 (2)	0.88	0.68–0.72	12.3 (2)	12.3(2)	12.6 (2)	12.3 (3)	11.9 (2)	12.2 (3)	12.3 (2)	12.3 (2)
Delivered nursing care	5	12.5 (3)	0.83	0.61–0.71	12.6 (3)	12.4 (2)	13.0 (2)	12.1 (3)	12.4 (2)	12.5 (3)	12.5 (3)	12.5 (2)
Nursing care innovation, administration, & management	6	17.2 (3)	0.86	0.67–0.70	17.3 (3)	17.1 (2)	17.8 (3)	17.2 (2)	16.7 (2)	16.2 (2)	17.1 (2)	18.4 (3)
Techno-medical care	6	16.8 (3)	0.84	0.60–0.70	17.1 (3)	16.5 (3)	17.0 (3)	16.8 (2)	16.6 (3)	16.8 (3)	16.8 (3)	16.8 (3)
Nursing care documentation & administration	8	21.9 (4)	0.89	0.69–0.78	22.5 (4)	21.2 (4)	24.4 (4)	21.0 (4)	20.2 (4)	19.4 (4)	20.9 (4)	25.3 (4)
Pedagogy in nursing care	5	12.9 (2)	0.87	0.70–0.78	13.3 (2)	12.5 (2)	13.5 (2)	12.5 (2)	12.7 (2)	12.9 (2)	12.9 (2)	12.9 (2)
NPCS-SV (overall)	35	80.4 (9)	0.89	-	77.9 (9)	82.8 (8)	85.6 (9)	78.5 (8)	76.9 (8)	77.9 (9)	78.8 (8)	84.3 (8)

**Table 2:** Rotated Factor Analysis of 33-items NPC-SV-A (*n* = 518).

Factor (Label)	1 (Nursing Care Administration & Documentation)		2 (Techno-Medical Care)		3 (Nursing Care Innovation, Administration, & Management)		4 (Value-Centered Nursing Care)		5 (Pedagogy in Nursing Care)		6 (Delivered Nursing Care)	
Item/ Loading	Item No.	Loading	Item No.	Item No.	Loading	Loading	Item No.	Loading	Item No.	Loading	Item No.	Loading
	22 23 24 25 26 27 28 29	0.812 0.809 0.821 0.783 0.748 0.697 0.871 0.761	11 12 13 14 15 16	0.752 0.864 0.754 0.850 0.695 0.801	30 31 32 33 34 35	0.791 0.842 0.683 0.814 0.687 0.834	6 7 8 9 10	0.810 0.768 0.787 0.726 0.794	17 18 20 21	0.803 0.762 0.757 0.798	1 2 4 5	0.745 0.638 0.775 0.782
Eigenvalue	14.87		11.35		9.68		8.68		7.26		4.37	
Variance explained	15.91%		14.02%		%12.65		10.77%		8.35%		5.82%	
Total Variance 67.52%												

Note: Item 3 and 19 was deleted in the preliminary EFA analysis.

**Table 3:** Inter-correlations, convergent, and discriminant validity measures of NPC-SV-A Subscales (*n* = 518).

No.	Subscales	1	2	3	4	5	6	CR	AVE	MSV	ASV
1	Nursing care administration & documentation	**0.732**						0.830	0.535	0.178	0.149
2	Techno-medical care	0.35 **	**0.751**					0.853	0.564	0.232	0.131
3	Nursing care innovation, administration, & management	0.23 *	0.37 **	**0.710**				0.815	0.504	0.384	0.164
4	Value-centered nursing care	0.41 **	0.28 **	0.48 **	**0.725**			0.865	0.526	0.384	0.257
5	Pedagogy in nursing care	0.36 **	0.27 **	0.16 *	0.47 **	**0.748**		0.826	0.559	0.363	0.219
6	Delivered nursing care	0.43 **	0.18 *	0.36 **	0.49 **	0.45 **	**0.737**	0.842	0.543	0.341	0.127

CR, composite reliability; AVE, average variance extracted; MSV, maximum shared variance; ASV, average shared variance, Square root of AVE in bold on diagonals * *p* < 0.01; ** *p* < 0.001 (two-tailed).

## Data Availability

The corresponding author will provide the datasets used in the current work upon reasonable request.

## Appendix A

**Table A1:** Summery of Scale Items Translation and Cultural Adaptation.

	Original Items	Culturally Adapted Back-Translated Items	Culturally Adapted Arabic Items
Delivered nursing care, 5 items			
1	Independently apply the nursing process	Apply the nursing process independently	تطبيق عملية التمريض بشكل مستقل
2	Meet patient’s basic physical needs	Satisfy the basic physical needs of the patient	تلبية الاحتياجات الجسدية الأساسية للمريض
3	Meet patient’s specific physical needs	Satisfying the patient’s confidential physical needs	تلبية الاحتياجات الجسدية السرية للمريض
4	Document patient’s physical status	Document the patient’s physical condition	توثيق الحالة الجسدية للمريض
5	Document patient’s psychological status	Documenting the patient’s psychological state	توثيق الحالة النفسية للمريض
Value-centered nursing care, 5 items			
6	Respectfully communicate with patients, relatives and staff	Communicate respectfully with patients, relatives, and staff	التواصل باحترام مع المرضى والأقارب والموظفين
7	Show respect for patient autonomy, integrity and dignity	Show respect for the patient’s independence, integrity and dignity	أظهر الاحترام لاستقلالية المريض ونزاهته وكرامته
8	Enhance patients’ and relatives’ knowledge and experiences	Enhance knowledge and experiences of patients and relatives	تعزيز معرفة وخبرات المرضى والأقارب
9	Show respect for different values and beliefs	Show respect for cultural values and beliefs	أظهر الاحترام للقيم والمعتقدات الثقافية
10	Contribute to a holistic view of the patient	Contribute to a comprehensive view of the patient	المساهمة في رؤية شاملة للمريض
Techno-Medical care, 6 items			
11	Manage drugs and clinical application of knowledge in pharmacology	Drug administration and the clinical application of knowledge in pharmacology	إدارة الأدوية والتطبيق السريري للمعرفة في علم العقاقير
12	Independently administer prescriptions	Independently manage medication prescriptions	إدارة الوصفات الطبية بشكل مستقل
13	Pose questions about unclear instructions	Ask questions about unclear instructions	اطرح أسئلة حول التعليمات غير الواضحة
14	Support patients during examinations and treatments	Supporting patients during examinations and treatments	دعم المرضى أثناء الفحوصات والعلاجات
15	Follow up on patient’s conditions after examinations and treatments	Follow up the patient’s condition after examinations and treatments	متابعة حالة المريض بعد الفحوصات والعلاجات
16	Handle medical/technical equipment according to legislation and safety routines	Handle medical/technical equipment in accordance with legislation and safety procedures	تعامل مع المعدات الطبية/التقنية وفقًا للتشريعات وإجراءات السلامة
Pedagogy in nursing care, 5 items			
17	Provide patients and relatives with support to enhance participation in patient care	Provide support to patients and enhance family to participate in patient care	تقديم الدعم للمرضى وتعزيز مشاركة العائلة في رعاية المريض
18	Inform and educate individual patients and relatives	Inform and educate patients and families	إعلام وتثقيف المرضى والعائلة
19	Inform and educate groups of patients and relatives	Inform and educate patient groups and families	إعلام وتثقيف مجموعات المرضى والعوائل
20	Make sure that information given to the patient is understood	Ensure that information given to the patient is understood	تأكد من أن المعلومات المقدمة للمريض مفهومة
21	Motivate the patient to adhere to treatments	Motivate the patient to adhere to the treatments	تحفيز المريض على الالتزام بالعلاجات
Nursing care documentation & administration, 8 items			
22	Make use of relevant data in patient records	Take advantage of relevant data in patient records	استفد من البيانات ذات الصلة في سجلات المرضى
23	Use information technology as a support in nursing care	Use of information technology as a support in nursing care	استخدام تكنولوجيا المعلومات كدعم في الرعاية التمريضية
24	Document according to current legislation	Document in accordance with country legislation	القيام بالتوثيق وفقًا لتشريعات الدولة
25	Comply with current legislation and routines	Comply with country legislation and guidelines	الامتثال لتشريعات الدولة والمبادئ التوجيهية
26	Handle sensitive personal data in a safe way	Treat sensitive personal data confidentially	تعامل مع البيانات الشخصية الحساسة بسرية
27	Observe work-related risks and prevent them	Monitor and prevent work-related risks	مراقبة المخاطر المتعلقة بالعمل ومنعها
28	Continuously engage in professional development	Continuously engage in professional development	الانخراط باستمرار في التطوير المهني
29	Lead and develop health staff teams	Leading and developing teams of health workers	قيادة وتطوير فرق العاملين الصحيين
Nursing care innovation, administration, & management, 6 items			
30	Act adequately in the event of unprofessional conduct among employees	Act appropriately in the event of unprofessional behaviors among employees	التصرف بشكل مناسب في حالة السلوك غير المهني بين الموظفين
31	Apply principles of disaster medicine	Apply the principles of disaster medicine	تطبيق مبادئ طب الكوارث
32	Search and review relevant literature for evidence-based nursing	Search and review relevant literature for evidence-based nursing	بحث ومراجعة الأدبيات ذات الصلة للتمريض القائم على الأدلة
33	Interact with other professionals in care pathways	Interact with other professionals in the care pathways	التفاعل مع المهنيين الآخرين في مسارات الرعاية
34	Teach, supervise and assess students	Teach, supervise and assess students	قم بتدريس الطلاب والإشراف عليهم وتقييمهم
35	Supervise and educate staff	Supervision and education of employees	الإشراف وتثقيف الموظفين
